# Patients’ perspectives on the use of artificial intelligence in dentistry: a regional survey

**DOI:** 10.1186/s13005-023-00368-z

**Published:** 2023-06-22

**Authors:** Nasim Ayad, Falk Schwendicke, Joachim Krois, Stefanie van den Bosch, Stefaan Bergé, Lauren Bohner, Marcel Hanisch, Shankeeth Vinayahalingam

**Affiliations:** 1 Department of Oral and Maxillofacial Surgery, Hospital University Münster, 48149 Münster, Germany; 2 Department of Oral Diagnostics and Digital Health and Health Services Research, Charité—Universitätsmedizin Berlin, Corporate Member of Freie Universität Berlin and Humboldt-Universität Zu Berlin, Aßmannshauser Str. 4-6, 14197 Berlin, Germany; 3 Department of Oral and Maxillofacial Surgery, Radboud University Nijmegen Medical Centre, P.O. Box 9101, 6500 HB Nijmegen, the Netherlands

**Keywords:** Artificial intelligence, Machine learning, Qualitative research, Patient survey, Perception

## Abstract

The use of artificial intelligence (AI) in dentistry is rapidly evolving and could play a major role in a variety of dental fields. This study assessed patients’ perceptions and expectations regarding AI use in dentistry. An 18-item questionnaire survey focused on demographics, expectancy, accountability, trust, interaction, advantages and disadvantages was responded to by 330 patients; 265 completed questionnaires were included in this study. Frequencies and differences between age groups were analysed using a two-sided chi-squared or Fisher’s exact tests with Monte Carlo approximation. Patients’ perceived top three disadvantages of AI use in dentistry were (1) the impact on workforce needs (37.7%), (2) new challenges on doctor–patient relationships (36.2%) and (3) increased dental care costs (31.7%). Major expected advantages were improved diagnostic confidence (60.8%), time reduction (48.3%) and more personalised and evidencebased disease management (43.0%). Most patients expected AI to be part of the dental workflow in 1–5 (42.3%) or 5–10 (46.8%) years. Older patients (> 35 years) expected higher AI performance standards than younger patients (18–35 years) (*p* < 0.05). Overall, patients showed a positive attitude towards AI in dentistry. Understanding patients’ perceptions may allow professionals to shape AI-driven dentistry in the future.

## Introduction

Recent advances in artificial intelligence (AI) have led to tremendous changes and challenges in the field of dentistry [1]. AI describes the theorisation and development of computer systems (e.g., neural networks) aiming to imitate the human intellect [2]. These networks are composed of many layers that transform input data (e.g., images) into outputs (e.g., disease presence/absence), allowing a wide range of possible applications from automated treatment planning to improved diagnostics [3].

In the near future, AI could play a major role in nearly every part of dentistry, including oral and maxillofacial surgery, cariology and endodontics, periodontics, paediatric dentistry, orthodontics and prosthodontics [2].

The image analysis of dental X-rays could be considered as the most important possible function of AI in dentistry. The frequent use of intraoral and panoramic images underlines the importance of the imaging sector in dentistry. Recently, AI has shown promising results in the analysis of dental X-rays in various studies [4–7]. The first Food and drug Adminisration (FDA) approved/ Conformité Européenne (CE) marked AI solutions are currently entering the market and the clinical arena [8].

Nevertheless, a range of challenges still require solutions. The technical complexity of modelling patient data to tailor medical decisions increases exponentially with the confronted uncertainties and erroneous data used in clinical decision making [9]. The limited availability of data, poor methodological reproducibility and the narrow usability of current AI systems are the three main reasons why AI has not yet become a major assistive technological system in dentistry [10]. Moreover, ethical and legal challenges remain; debates around data privacy, safety and effectiveness or liability are still ongoing. Open public and political discussions are desired to rethink the current regulatory frameworks and ensure they can be adapted to the healthcare system [11]. However, the extent to which AI solutions are adapted in dentistry will mainly depend upon the attitudes of clinicians and patients [12].

Few studies have investigated dentists’ and dental students’ attitudes and perceptions towards AI [13–15]. These studies shared optimistic views, with a positive impact on dental care. One recent controlled study assessed patients’ perspectives towards AI for radiographic caries detection and the impact of AI-based diagnosis on patients’ trust [16]. Compared to Kosan et al., the current work addressed patients´ perspectives on AI in general.

Another study assessed patients’ perceptions of the use of AI in radiology and identified six key domains of patients’ perspectives: (1) proof of technology, (2) procedural knowledge, (3) competence, (4) efficiency, (5) personal interaction and (6) accountability [17].

The purpose of the present study was to assess patients’ knowledge and perceptions of AI in dentistry. This study was purely explorative, without concrete hypothesis testing. Understanding patients’ perceptions may allow dentists to shape AI-driven dentistry in the future.

## Materials and methods

A modified 18-item questionnaire in the German language (Table 1) was used based on the survey design by Scheetz et al. [12]. The survey questions focused on demographics, self-perceived dental health, expectancy, accountability, trust, interaction, advantages and disadvantages. The survey questions were validated through a literature review and pilot testing. Disagreements regarding any questions were resolved by consensus (NA, LB, MH). To avoid any potential discrimination, questions regarding gender or education were omitted. The inclusion criteria were a minimum age of 18 and fluent proficiency in German. Approval for this survey was granted by the Ethics Committee of the Westphalia-Lippe Medical Association, Westfälische-Wilhelms University Münster (IRB approval no. 2021–616-f-S). This study was conducted in accordance with the code of ethics of the World Medical Association (Declaration of Helsinki).

**Table 1:** Questionaire

**How old are you?**										
☐18–25		☐ 26–35			☐ 36–50				☐ 51–60	☐ 61–80
**How often do you visit the dentist per year?**										
☐ semiannual		☐ annual			☐ biennial				☐ emergency	
**How important is your oral health to you?**										
☐ not at all		☐ low			☐ normal				☐ high	
**Which dental treatments had you in the past? (Multiple choice possible)**										
☐ Fillings ☐ Root canal treatment ☐ Bleaching		☐ Prosthetics (crown, bridge, prothesis etc.) ☐ Apicectomy			☐ Tooth extraction ☐ None ☐ Other: ______________________________					
**Have you had panoramic radiographs/ radiographs taken in the past?**										
☐ yes					☐ no					
**How would you assess your knowledge about the digital world?**										
☐ nothing		☐ below average			☐ average					☐ above average
**How would you assess your knowledge about artificial intelligence?**										
☐ nothing		☐ below average			☐ average					☐ above average
**Do you have concerns about the use of artificial intelligence in dentistry? If yes, which? (Multiple choice possible)**										
☐ No concerns ☐ Impact on workforce needs ☐ Data security ☐ Lack of accuracy of “electronic” diagnoses		☐ New challenges for the doctor-patient-relationship ☐ Costs ☐ The influence of technology giants ☐ Other concerns:____________________								
**From your point of view, what is the greatest advantage of using artificial intelligence in dentistry? (Multiple choice possible)**										
☐ Time savings ☐ Better doctor-patient-relationship ☐ More safety in diagnoses ☐ Increase in treatment quality		☐ Improved health comprehension ☐ More uniformity among dentists ☐ Other: _____________________________________								
**Would the use of artificial intelligence be able to compensate mistakes from the dentists?**										
☐ no		☐ yes			☐ unsure					
**Do you think that the use of artificial intelligence would lead to shorter treatment time?**										
☐ no		☐ yes			☐ unsure					
**Does the use of artificial intelligence improve the overall oral health?**										
☐ no improvement		☐ less improvement			☐ yes					☐ major improvement
**Do you think that the use of artificial intelligence would lead to an increase the treatment quality?**										
☐ no improvement		☐ less improvement			☐ yes			☐ major improvement		☐ no opinion
**Does the use of artificial intelligence improve the doctor-patient-relationship?**										
☐ no improvement		☐ less improvement			☐ yes			☐ major improvement		☐ no opinion
**What level of error is tolerable for artificial intelligence-based diagnostic systems?**										
☐ Equivalent to the worst performing dentist		☐ Equivalent to the average performing dentist			☐ Superior to the average performing dentist			☐ Equivalent to the best performing dentist		☐ Superior to the best performing dentist
**Would you endorse the following procedure? Dental X-rays will be analyzed by an artificial intelligence and the findings will be assessed by the dentist afterwards**										
☐ yes	☐ no					☐ unsure				
**To what extent would you agree to the following statement? Dentistry will benefit from the use of artificial intelligence**										
☐ yes	☐ no			☐ unsure						
**When would artificial intelligence be fully implemented in the daily dental work?**										
☐ within one year		☐ in 1 to 5 years	☐ in 5 to 10 years				☐ in over 10 years			☐ never

### Sample size

Sample size estimation and power calculations were waived by the Institute of Biostatistics and Clinical Research of Westfälische-Wilhelms University Münster. This study was purely explorative and observational, using convenience sampling without concrete hypothesis testing. 

### Study design

The cross-sectional questionnaire was distributed to patients who visited the outpatient clinic at the Department of Cranio-Maxillofacial Surgery, University hospital Münster, and a private dental clinic in Münster, Germany, between November 2021 and March 2022. These two clinics cover a broad range of clinical areas, such as implantology, oral surgery, reconstructive dentistry, prosthodontics, orthognathic surgery and periodontics. Participation was voluntary, and no incentives were provided. Written in-formed consent was obtained from each participant prior to the survey commencement.

All participating patients received an ethically approved information sheet with a written explanation of the anticipated use of AI in dentistry (e.g., intraoral scan segmentation and digital implant position design). For further clarity, the first AI application receiving CE marking in dentistry (dentalXrai Pro, dentalXrai GmbH, Berlin) was provided as an example.

Descriptive statistical analysis was conducted using SPSS Statistics Version 28.0 (IBM, Armonk, NY, USA). Depending on the frequency, group differences were calculated using two-sided chi-squared or two-sided Fisher’s exact tests. An asymptomatic approximation using the Monte Carlo method was used in cases with invalid computational times. The level of significance was set at *p* < 0.05.

## Results

**Fig. 1:**
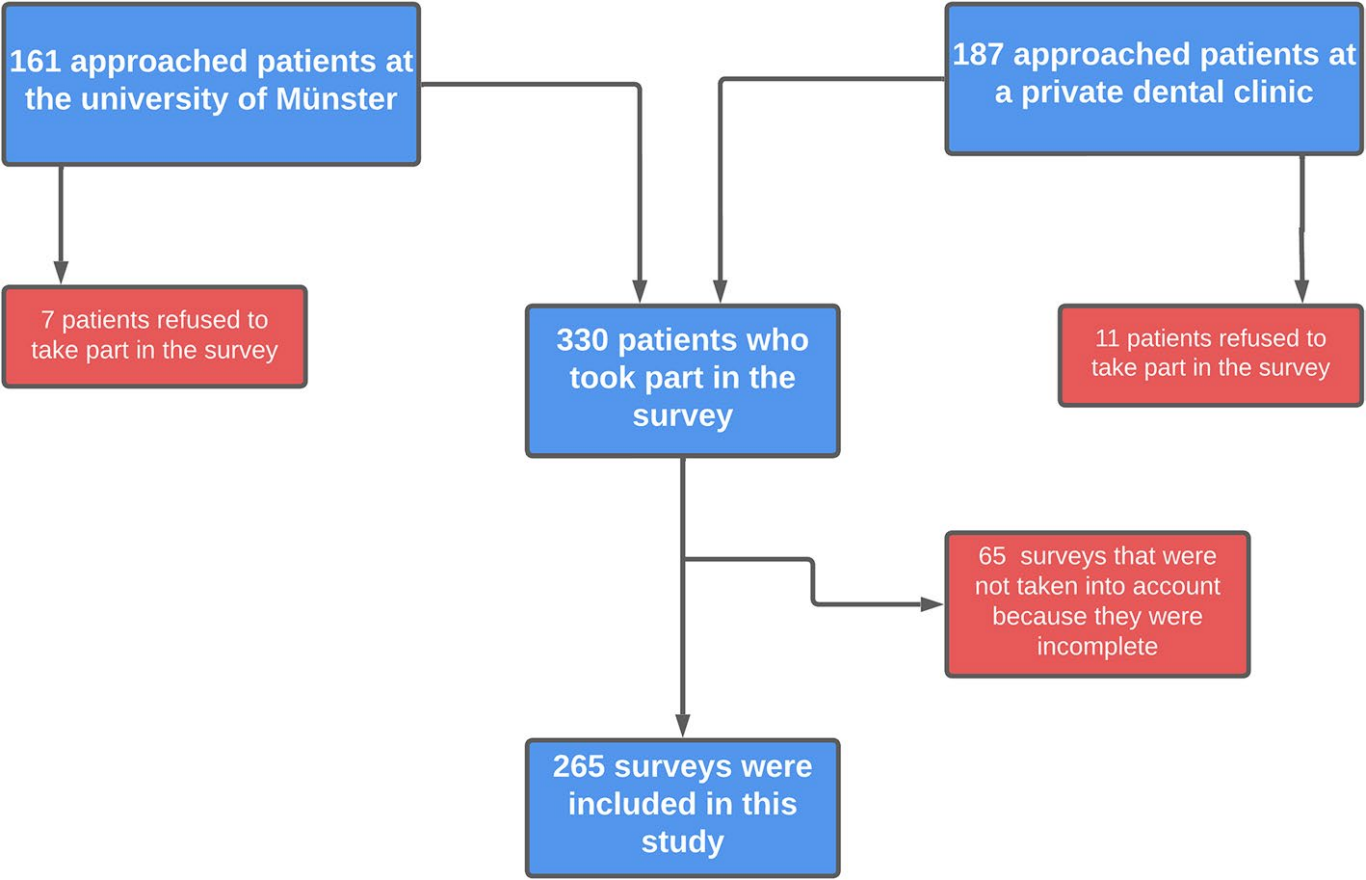
Flowchart showing the survey distribution procedure

Of the 348 participants to whom the questionnaire was distributed, 18 (5.2%) refused to answer, and 265 answered the questionnaire completely. Sixty-five incomplete surveys were excluded from further analysis based on the exclusion criteria. The flowchart showing the survey distribution procedure is shown in Fig. 1.

The demographics and self-perceived dental health of the 265 patients are outlined in Table 2.

**Table 2:** Demographics and self-perceived dental health

		***N* (%)**
**Age**	18–25	28.7
	26–35	23.0
	36–50	16.6
	51–60	17.0
	61–80	14.7
**Regular Dental Check Up**	Semiannual	54.0
	Annual	34.7
	Biennial	4.5
	Emergency	6.8
**Individual Importance of Oral Health**	Low	0.8
	Normal	33.2
	High	66.0
**Received Treatments in the Past**	Filling	81.5
	Prosthodontics	44.5
	Extraction	42.6
	Root canal treatment	38.9
	Bleaching	8.3
	Apicectomy	22.3
	Other	17.0
	None	3.8

### Self-perceived knowledge of artificial intelligence

The majority of the patients (*n* = 248, 93.6%) assessed their knowledge of digital technology as ‘average’ or ‘above average’. In comparison, only half of the participants (*n* = 139, 52.5%) rated their knowledge of AI as ‘average’ or ‘above average’. In total, 47.5% (*n* = 126) rated their knowledge of AI as ‘nothing’ or ‘below average’.

### Disadvantages of using AI in dentistry

**Fig. 2:**
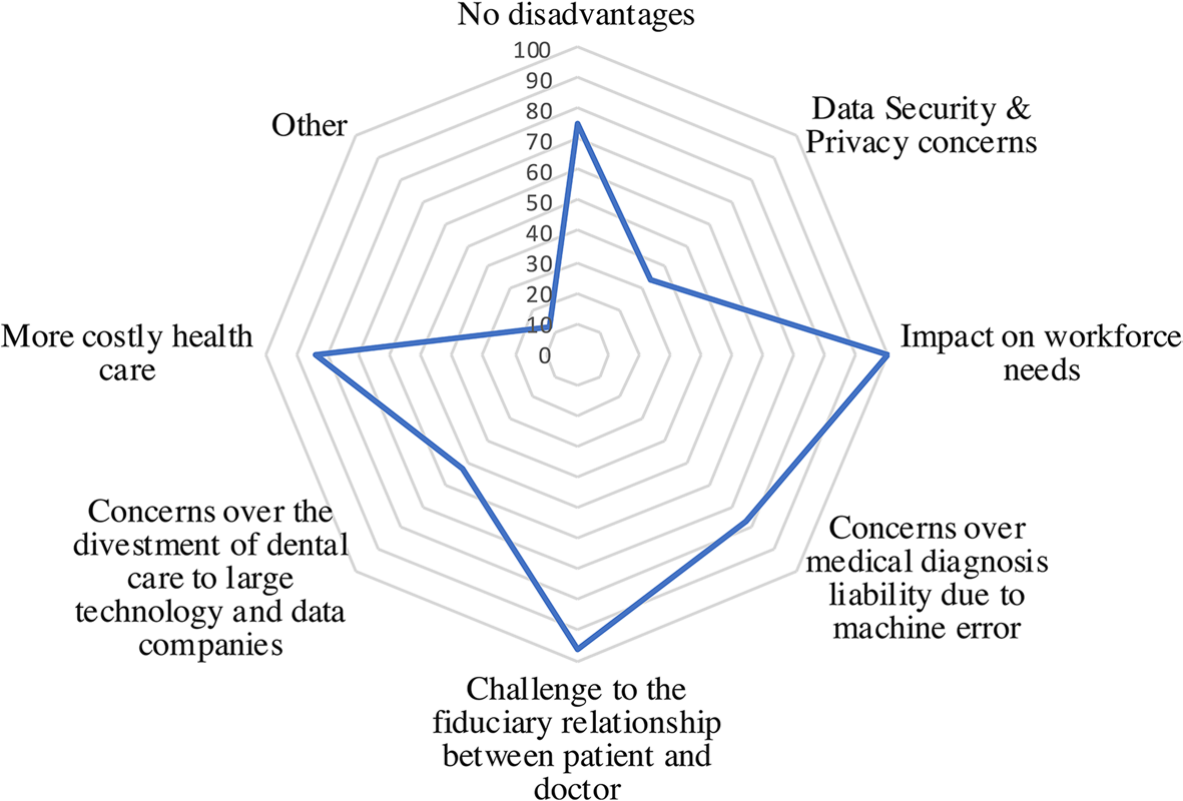
Cited Disadvantages of using AI in dentistry

The top three disadvantages cited by patients regarding the use of AI in dentistry were (1) the impact on workforce needs (37.7%), (2) new challenges to the fiduciary relationship between dentist and patient (36.2%) and (3) increased dental care costs (31.7%) (Fig. 2). Additional expressed disadvantages were liability, neglect of education, limited applicability for older dentists and lack of empathy. Only 12.8% of the cases were perceived as having a data privacy disadvantage.

### Major advantages of AI in dentistry

**Fig. 3:**
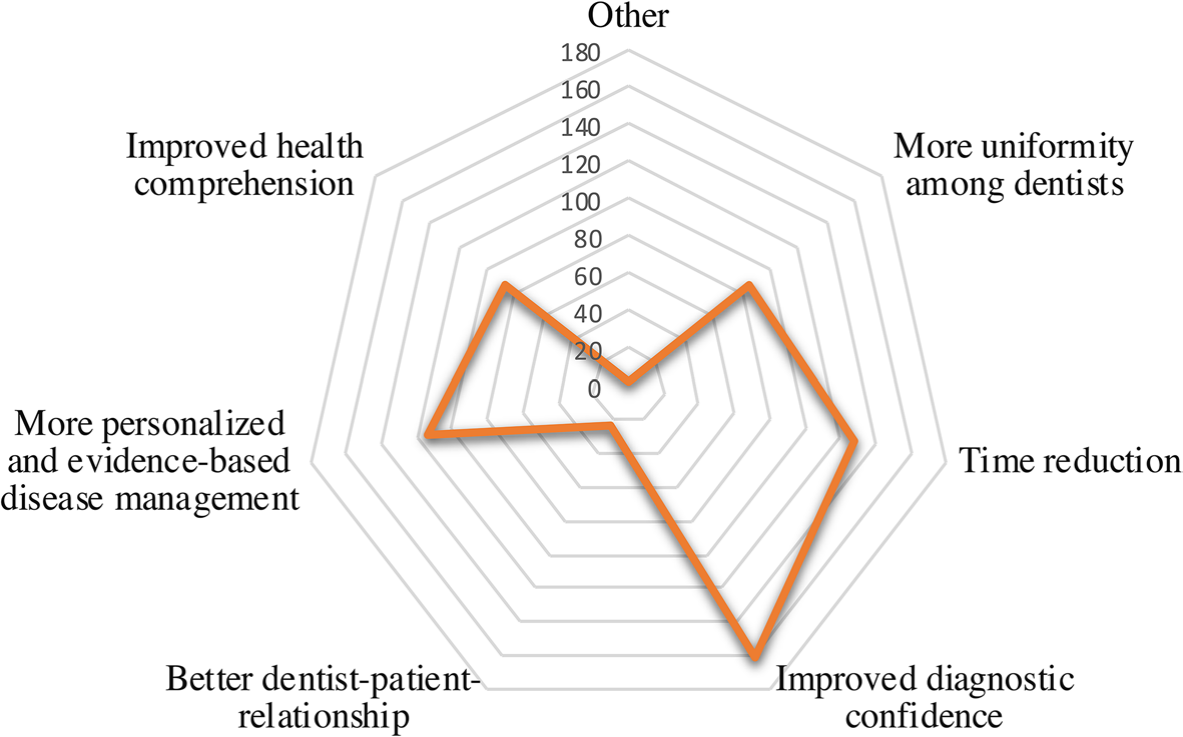
Cited Advantages of using AI in dentistry

The top three advantages respondents perceived regarding the use of AI in dentistry were ‘improved diagnostic confidence’ (60.8%), followed by ‘time reduction’ (48.3%) and ‘more personalised and evidencebased disease management’ (43.0%) (Fig. 3). Another stated advantage was the dual-control principle. The dual-control-principle is a theory that requires for certain activities (i. e. decision making in diagnosing or treatment) at least two operators/systems in order to increase accuracy and transparency. In addition, the impact on the fiduciary relationship between the dentist and patient was considered an advantage by only 9.1% of respondents.

### Improvement in public oral health

Regarding whether the implementation of AI would lead to better public oral health, the majority (*n* = 137, 51.7%) responded that they were unsure. About one-quarter of the volunteers (*n* = 73, 27.5%) believed that public oral health would experience a major improvement due to the use of AI.

### Hypothetical workflow for diagnosing dental x-rays

One possible future way of diagnosing intra and extraoral dental images could be as follows: Dental images will be analysed by an AI first and the findings will be assessed by the dentist afterwards. Of the respondents, 84.9% (*n* = 225) agreed with this proposed procedure, while a minority (4.9%, *n* = 13) disagreed.

### Acceptable AI performance standards and clinical workflows

Around half of the patients (*n* = 125, 47.2%) stated that AI should perform better than an average-performing dentist, while 29.1% (*n* = 77) expected AI to be as good as the best dentist. Only a minority (7.5%, *n* = 20) demanded that AI be better than the best performing dentist. Significant differences were seen between younger (18–35 years) and older (> 35 years) patients in terms of acceptable AI performance standards (*p* < 0.05). Older patients expected higher AI performance standards. Around half of the respondents assumed that the use of AI could prevent mistakes made by dentists (*n* = 129, 48.7%).

### Expected time until AI will be part of dental workflows

Most patients expected that AI would be part of the dental workflows either in 1–5 (*n* = 112, 42.3%) or 5–10 years (*n* = 124, 46.8%) years. Only a tenth (*n* = 21, 7.9%) expected AI integration 10 years or later. Furthermore, the majority (*n* = 195, 73.6%) agreed with the following statement: ‘Dentistry will benefit from the use of artificial intelligence’.

## Discussion

The use of AI in dentistry is rapidly evolving and could play a major role in the dental office in the near future. As Chen et al. [18] pointed out, AI systems can be categorised into pre-, inter- and post-appointment AI systems. The idea of a comprehensive AI dental care system predicts that AI could play a role in patient management (pre-appointment) to analyse patients’ needs and risks, in diagnosis and treatment planning/decision and in outcome prediction (inter-appointment), as well as in labour work (e.g., design for prosthodontics) and treatment evaluation (post-appointment). This model emphasises the variety of tasks that AI could take part in. Nevertheless, the inter-appointment AI systems would be most visible to patients, as these systems would intervene in diagnosis, treatment decisions and planning.

The present survey assessed patients’ perceptions and attitudes towards AI in dentistry. Our findings are in line with previous surveys. The introduction of new technologies will lead to a shift in skill and expertise requirements. A consensus has arisen that education and training programmes need to be adjusted to labour market requirements [19]. Furthermore, surveys have highlighted disadvantages regarding employment prospects [20, 21]. However, as other studies have pointed out, AI will rather work synergistically with clinicians than replace clinicians completely by overtaking clinical work [22]. The conclusion of other surveys that investigated the attitudes of dental students towards AI was that implementing AI in further dental training curricula is important [15, 23].

AI-based communication often lacks intentionality and therefore constitutes a significant obstacle at the communication level. In line with these findings, previous studies have shown a preference for clinician based diagnostic decisions over AI-based diagnostic decisions [17, 24–26]. Handing over parts of the communication to AI-driven systems as part of the diagnosis and treatment procedure can be crucial to the wellbeing of the patients and therefore the relationship between dentists and patients. Compared with the findings of this study, about a third of the participants stated that the use of AI in the dental field could arise new challenges to the fiduciary relationship between dentist and patient (36.2%). However, assistive AI systems should provide support to the dentist, which would enhance their ability to attend to higher valued tasks, such as more professional interactions with patients, integrating patients into the diagnosis and treatment process and being more visible to the clinical team and patients [27]. The dentist is constantly asked to nonverbally assess the patient’s feelings and adapt their further actions based on their evaluation [28]. Moreover, due to the fact that 330 patients took part in the survey and only eighteen patients have declined to do so, an enormous interest for AI in dentistry among dental patients is observable. This underlines the importance of such surveys in order to demonstrate the possibilities of AI implementation in the dental field for patients.

Lastly, AI-based dental care will raise new questions about cost-effectiveness and ever rising healthcare costs. About a third of the patients, that have participated in our survey concern about a possible raise of costs. However, Rossi et al. analysed cost-effectiveness using health economic modelling via Markov models and concluded that the current evidence supporting AI as a decision support mechanism is limited [29, 30]. More investigation is needed.

Previous studies have referred to AI as a facilitator of faster, more precise and more personalised and evidence-based disease management. These studies have reported on par or even higher diagnostic accuracies than average dentists [8, 10, 31].

To be more precise, AI is mainly used in lesion segmentation in the fields of cariology and endodontics. Identifying tooth surface loss, root caries, periapical lesions or root fractures is crucial in further treatment decision-making. Depending on the diagnosis, the affected tooth may undergo tooth preserving/restorative treatment or, in the worst case, extraction. The treatment decision will have a major impact on further treatment requirements. Accurate and precise detection of lesions is important in the implementation of suitable treatments [32]. In terms of caries detection, numerous studies have reported that trained and validated AI systems have significantly higher accuracy and sensitivity than clinicians [8, 29, 32, 33]. As the present study assessed the acceptable performance of AI, about half of the participant stated the AI systems should be better than an average dentist and could prevent mistakes made by the dentist. Compared with the studies that investigated the actual performance of AI, current AI-systems could live up the participants´ claim.

The detection of periapical lesions in endodontic treatment is essential for further treatment planning and the prediction of treatment success. Setzer et al. reported excellent results in identifying periapical lesions on Cone Beam Computer Tomography (CBCT) using a deep learning system based on an encoder-decoder architecture (U-NET) [34]. The application of such systems could lead to more reliability in diagnosis, with accuracy levels on par or even higher than experienced specialists [35].

Furthermore, the development of AI systems in the fields of oral and maxillofacial surgery is promising. AI systems may help in diagnosing the pathologies of various types of diseases and assist in treatment planning. Neural networks may be used to diagnose pathologies in the bone and mucosa, guiding orthognathic surgery, implantology and complication management [36]. For example, with an accuracy of 94%, a support vector machine (SVM) classifier system was able to differentiate between periapical cysts and keratocystic odontogenic tumours in CBCT scans [37]. In addition, diagnosing tumorous lesions at an early stage has a significant impact on disease treatment and outcomes. AI systems may have the ability to help professionals and mitigate healthcare delays [38]. In terms of orthognathic surgery, AI could play a major role from treatment planning to treatment follow-up. With the ability to visualise the treatment beforehand, confusion and misunderstanding by the patient may be limited [39].

In orthodontic treatment planning, the analysis of cephalometric radiographs is vital to successful treatments. The automated detection of landmarks by a knowledge-based algorithm showed no significant difference from manual detection [40, 41]. Another study showed that a web-based deep learning application analysing 23 landmarks on cephalograms showed a classification success rate of 88.43% [42]. Camci et al. showed that a deep learning system that evaluated the tooth size of unerupted teeth had higher accuracy than the use of a Moyer’s table [43]. A scoping review stated the possibility of improving diagnostic accuracy in orthodontics using AI [44].

AI can also be used in paediatric dentistry. Numbering deciduous teeth in paediatric panoramic radiographs is crucial to identifying congenital or non-congenital issues. Recently, a convolutional neural network (CNN)-based system was able to detect deciduous teeth in panoramic radiographs with a sensitivity of 0.98 [45].

As the majority of the patients have stated that AI could lead to an improved diagnostic confidence, the opinion of most participants comes in the line with the studies that observed promising results regarding AI performance in the dental field. When it comes to an improved public oral health by using AI in dentistry, only about a quarter of the participants expect a major improvement. The authors conclude that more studies are needed to create awareness for AI-systems among patients.

Nevertheless, these models need to undergo a more transparent validation process using external data to verify their generalisability and reliability [46]. Due to the rapid development process, AI systems may be considered black-box systems by critical users and consumers. Developers may need to stress the principles of these models in a more transparent manner to keep clinicians and patients engaged and to avoid trust issues [47]. As about half of the respondents (47.5%, *n* = 126) rated their knowledge of AI as ‘nothing’ or ‘below average’, integrating patients in the development process and creating greater insight into the medical AI world may lead to enhanced AI knowledge. Transparency in the developing process could lead to more trustworthiness among patients.

Furthermore, AI models have proven to be extremely time-efficient for certain tasks, such as image segmentation [6]. Yasa et al. used a CNN-based system to identify teeth in bitewing radiographs, which yielded promising results in terms of accuracy and time efficiency [48]. Improving the dental workflow and increasing diagnostic accuracy may lead to more efficient, personalised dentistry. Time-efficiency have also been stated to be a major advantage of using AI in dentistry by the participants of the study.

The present study has a range of strengths and limitations. First, it is one of the few studies that focused on the attitudes and expectations of patients towards AI in dental and maxillofacial fields. The representativeness of this explorative study should be regarded with caution for several reasons. Even though the sample size was reasonable, larger sample sizes are needed for in-depth subgroup analysis next to validated instruments. Furthermore, Germany is a high-income country and therefore higher oral care awareness can be expected. Future studies can then be tested with a concrete hypothesis in reference to this study.

## Conclusion

Overall, patients showed a positive attitude towards the use of AI in dentistry. Major advantages were the increase in diagnostic confidence, time efficiency and more personalised disease management. To achieve an increase in knowledge about AI, the development of AI should be more transparent and visible to patients.

## Data Availability

The data used in this study can be made available from the corresponding author within the regulation boundaries for data protection.
